# Emotional dysregulation as a moderating factor on the relationship between the severity of anxiety and depressive symptoms and high caregiving intensity among mothers caring for children diagnosed with life-limiting conditions

**DOI:** 10.1017/S1478951526102211

**Published:** 2026-04-13

**Authors:** Rufaida Hassan Ibdah, Fadi Zaben, Rozan Al-Sarayreh, Ayman Hamdan-Mansour

**Affiliations:** 1 Department of Primary Care Nursing, Faculty of Nursing, Al-Ahliyya Amman University, Amman, Jordan; 2 Department of Pediatric Nursing, Faculty of Nursing, An-Najah National University, Nablus, Palestine; 3 Department of Pediatric Nursing, Faculty of Nursing, Al-Zaytoonah University of Jordan, Amman, Jordan; 4 School of Nursing, The University of Jordan, Amman, Jordan

**Keywords:** Anxiety, depression, emotional dysregulation, life-limiting conditions, caregiving burden

## Abstract

**Objective:**

Providing care for children with life-limiting conditions(LLCs) is an emotionally challenging experience that often exposes caregivers, particularly mothers, to considerable risk of psychological distress. The purpose of this study was to examine the moderating effect of emotional dysregulation on the relationship between severity of anxiety and depressive symptoms and high caregiving intensity, controlling for sociodemographic characteristics among mothers caring for children diagnosed with life-limiting conditions.

**Method:**

Using a cross-sectional descriptive design, a convenience sample of 192 mothers caring for children with life-limiting conditions was recruited and filled out an online self-administered questionnaire. Data were collected using online self-administered questionnaires regarding the sociodemographic characteristics of mothers and their children, emotional regulation difficulties (DERS), and the levels of anxiety and depressive symptoms among the mothers (DASS-21).

**Results:**

The analysis showed that 21.4% and 7.8% of mothers had moderate and severe depressive symptoms, and 19.3% and 15.6% had moderate and severe anxiety symptoms, respectively. The analysis also showed that emotional dysregulation is associated with high levels of anxiety (β = 0.74, *P* < 0.001) and depression (β = 0.74, *P* < 0.001); however, there was no significant moderating effect.

**Significance of results:**

Anxiety and depression are significant psychological distress among mothers caring for children with life-limiting conditions and can be aggravated by emotional dysregulation and caregiving burden. There is a need to integrate interdisciplinary teamwork and family-centered care to provide holistic care and offer early screening, detection, and emotional regulation-focused management programs for psychological distress at healthcare services that care for children with LLCs.

## Introduction

Life-limiting conditions (LLCs) are incurable disorders from which children, assumingly, will die (Fraser and Parslow 2017). Some LLCs may cause early death, while others may influence life expectancy, leading to death during childhood, adolescence, or early adulthood. LLCs encompass several categories of disorders, including neurodegenerative diseases, congenital anomalies, metabolic diseases, respiratory disorders, and immunodeficiency diseases (Fraser and Parslow 2017) . Caring for a child with LLCs involves extended and complex care that requires family caregivers to manage various physical, psychological, and socio-economic demands. It may include activities of daily living, community engagement, feeding, hygiene, dressing, medical care coordination, health decision-making, managing deterioration and uncertainties in the child’s health, and providing emotional support (Crowe et al. 2025). Such a complex and ongoing nature of caregiving is considered a traumatic experience for family members, which would contribute to chronic psychological distress, which has subsequently been associated with increased levels of anxiety and depression among caregivers, who are mostly mothers (Fisher et al. 2022). Several studies in Arabian societies showed that mothers are the primary caregivers for their children. This infers that mothers of children with LLCs are vulnerable to psychosocial and caregiving burdens and disturbances (Hafez et al. 2022).

Caregiving burden among mothers caring for children with LLCs involves significant actual or threatened resource losses, such as time, sleep, finances, energy, and social relationships. The Conservation of Resources (COR) theory posits that such losses compromise psychological resources, leading to emotional dysregulation and psychological distress (Hobfoll 1989; Hobfoll et al. 2018). Emotion regulation in Dialectical Behavior Therapy (DBT) is conceptualized as an individual’s ability to understand, accept, and modify emotional responses through techniques that decrease vulnerability to psychological distress (Linehan 1993; Neacsiu et al. 2010). DBT’s emotion regulation module involves skills such as mindfulness, distress tolerance, and adaptive emotional regulation (Neacsiu et al. 2014; Linehan 2015). On the other hand, emotional dysregulation is defined as difficulty monitoring, evaluating, and modulating emotional responses (Gratz and Roemer 2004). It is associated with more dependence on maladaptive coping, such as rumination and avoidance, which results in heightened vulnerability to psychological distress. For mothers caring for children with LLCs who are experiencing exhaustion from ongoing demands, emotional regulation skills become a challenging task during the ongoing care of children with LLCs (Lee et al. 2024). Therefore, the level of emotional dysregulation shapes emotional responses to stress, suggesting that the same caregiving burden could result in different levels of psychological distress depending on mothers’ emotional dysregulation levels.

Therefore, those mothers are at risk of developing severe forms of anxiety and depressive symptoms while providing ongoing care for their children (Xiong et al. 2020). Mahmoud et al. (2021) reflected that almost half of caregivers of children with chronic kidney disease, who are mostly mothers, experience moderate depression and severe anxiety and depressive symptoms. Consequently, Collins et al. (2020) revealed that half of the mothers of children with LLCs in their study met the criteria for one or more diagnoses of clinical anxiety and/or depression. Thus, the psychological resources of primary caregivers are a critical concern for both caregivers themselves and for healthcare professionals who assume that caregivers are responsible for ensuring the quality of physical and emotional care for children with LLC. Several mothers’ and children’s related factors were found to play a vital role in the severity of anxiety and depression among mothers, such as the duration of disease, the severity of disease symptoms, the required caregiving tasks, the mother’s level of education, and receiving social support (Su et al. 2022; Almulla et al. 2024).

This suggests that the psychological outcomes of caregiving burden are not always consistent and may be moderated by factors that may interfere with and moderate the severity of such psychological distress. Emotional regulation (ER) has been proposed in the literature to buffer anxiety and depressive symptoms among mothers (Panzeri et al. 2024) This may suggest that mothers caring for children with LLCs who had lower level of emotional dysregulation would be able to manage caregiving demand and effectively reduce the level of anxiety and depressive symptoms (Ruan et al. 2023; Shaffer et al. 2023). Acknowledging that mothers are the primary caregivers to their children, the above-mentioned discussion would draw attention to investigating emotional dysregulation as a critical moderator factor that intensifies the consequences of high caregiving intensity among these mothers. Therefore, the study’s genuine impact will be on the healthcare outcomes of the holistic treatment plans.

**Figure 1.**
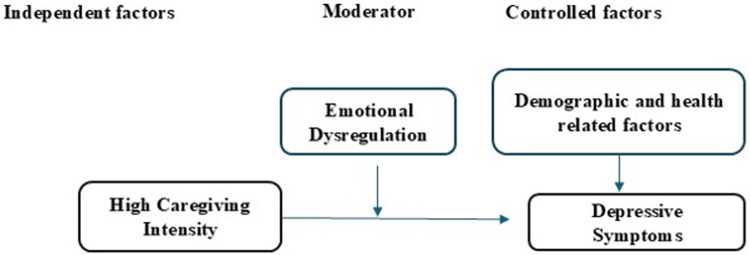
The moderation effect of emotional dysregulation on the relation between high caregiving intensity instensity and depressive symptoms.

**Figure 2.**
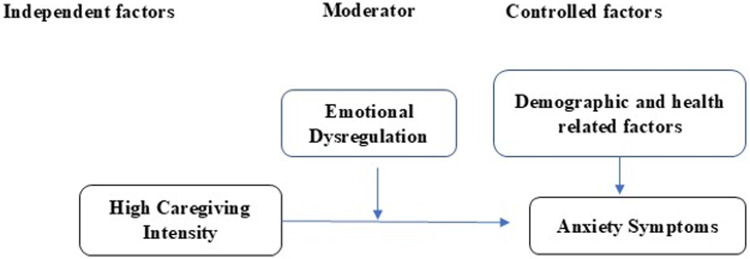
The moderation effect of emotional dysregulation on the relation between high caregiving intensity instensity and anxiety symptoms.

In Jordan, children with LLCs receive care across a fragmented medical system of specialties operated in separate facilities and hospitals. These healthcare services do not include any form of general supportive mental health or psychological care or specialized services. This would compromise the mental health status of mothers, which consequently affects negatively their ability to provide quality care to their children with LLCs. This study would be a pioneering, informative one that addresses the moderating role of emotional dysregulation in the relation between the high caregiving intensity, and the level of anxiety and depression among mothers caring for children with LLCs Figure 1 and 2. Therefore, the purpose of this study was to examine the moderating effect of emotional dysregulation on the relationship between severity of anxiety and depressive symptoms and high caregiving intensity, controlling for sociodemographic characteristics among mothers caring for children diagnosed with life-limiting conditions.

## Methods

**Design**: A cross-sectional descriptive correlation design was used. Data were collected using online self-administered questionnaires from mothers of children with LLCs. Data were collected regarding the emotional regulation difficulties and the levels of anxiety and depressive symptoms among mothers. Data were collected during the period March–June 2025.

**Setting**: This study was conducted in the pediatric outpatient clinics at 2 major public referral hospitals in the central district of Jordan. These pediatric clinics provide follow-up and specialized care for children diagnosed with various LLCs. The environment offers a suitable and accessible location for recruiting mothers who are actively involved in caregiving tasks.

**Sample and sampling**: Convenience sampling technique was employed to recruit mothers during their regular follow-up visit to the pediatric clinic. Inclusion criteria included: (1) Mothers currently caring for one or more children (1 > and <18) with LLCs who have had a diagnosis for at least 3 months, (2) able to read and write Arabic, (3) have access to smartphones and the WhatsApp platform. Mothers who are currently diagnosed with mental illness were excluded to reduce the potential confounding effect on the anxiety and depression symptoms.

## Data collection procedure

Data collection started after obtaining ethical approval from the ethical committee at the Ministry of Health (Ethics committee # ABM/3902). Pediatric physicians, after being informed about the study purpose and procedure, assisted in inviting mothers of children with LLCs. The pediatricians used Fraser et al.’s (2020) diagnostic coding framework and their clinical judgment. Eligible mothers were provided with comprehensive information regarding the study’s purpose, confidentiality, and procedures. Mothers were reassured that their participation was voluntary and that they had the right to refuse or withdraw at any time without any direct or indirect influence on their child’s provided quality of care. Interested mothers were then contacted via WhatsApp message and given a secure web link to the survey. The survey’s first page contained an informed consent, followed by demographic data for both mother and child, and then validated Arabic versions of DASS-21 and DERS-16. Access to the study instruments was restricted until electronic consent was obtained. A total of 249 mothers were recruited, with 192 completing the survey, resulting in a response rate of 77%. Data were collected anonymously and stored in password-protected, coded files to ensure confidentiality and adherence to ethical standards.

**Instruments**: All instruments in this study were used in their Arabic validated version.

1. Depression, Anxiety, and Stress Scale (DASS-21) (Lovibond and Lovibond 1995) was used to measure Depression and Anxiety. The Arabic version of the DASS-21 was used (Moussa et al. 2016). It is a self-report instrument that consists of 21 items, with 7 items in each subscale; each item was rated on a 4-point Likert scale. For the purpose of the study, the anxiety and depression subscales were only used. The scores of each sub-scale were calculated by summing the scores of each domain’s items and then multiplying by 2; elevated scores reflect an increase in symptom frequency and severity, with 5 severity levels categorizing: normal (0–9), mild (10–13), moderate (14–20), severe (21–27), and extremely severe (>28) for depression. Normal (0–7), mild (8–9), moderate (10–14), severe (15–19), and extremely severe (>20) for anxiety. The English version of the questionnaire has shown high internal consistency, with Cronbach’s alpha coefficients reported as 0.83 for depression, 0.79 for anxiety, and 0.81 for stress (Laranjeira et al. 2023). The Arabic version of DASS-21 has also been validated, reliable, and culturally appropriate, and has been used widely in studies (Moussa et al. 2016). DASS-21 has reported Cronbach’s α ranges from 0.83 to 0.94 across various Arabic populations (Ali et al. 2017; Alharbi and Osman 2023; Al–Dassean and Murad 2024). In the current study, internal consistency for the Anxiety and Depression subscales was calculated using Cronbach’s α
2. The Difficulty in Emotion Regulation Scale (DERS-16) (Bjureberg et al. 2015) was used to measure emotional regulation. The Arabic version of DERS-16 was used in this study (Fekih-Romdhane et al. 2023). DERS-16 is a brief self-reported instrument that consists of a 16-item scale rated on a 5-point Likert scale that assesses difficulties in emotion regulation. Higher scores reflect a higher level of emotion regulation difficulties. It is derived from the original DERS-36 and has strong psychometric properties while reducing the time of completion of the questionnaire. The Arabic version of DERS-16 has shown strong psychometric properties, with McDonald’s omega coefficients ranging from 0.81 to 0.95. (Fekih-Romdhane et al. 2023).
3. **High caregiving intensity:** High caregiving intensity was operationalized using 2 criteria: (1) providing 20 hours or more of direct caregiving tasks per week (Shattnawi et al. 2023), and (2) being the primary caregiver of 2 or more children diagnosed with life-limiting conditions (Aldas et al. 2025). Mothers self-reported the number of caring hours and the number of children via the demographic data required for the study survey. Mothers who have both criteria are considered to have a high caregiving intensity. These criteria were selected to identify high-intensity caregiving situations, which are clinically relevant for mothers managing the complex needs of children with life-limiting conditions. This method prioritizes practical signs of high caregiving intensity rather than subjective perception, confirming the inclusion of mothers with objectively demanding roles.

**Demographics:** An electronic data sheet was used to collect demographic and socio-economic data about mothers and their children. The mothers’ data included their age, marital status, physical health conditions, employment status, and educational level. The children’s data were about age, sex, duration of illness, admission numbers during the last year, caring hours, having more than one child diagnosed with LLC, and the cost of treatment.

## Data analysis

Data were analyzed using the Statistical Package for Social Sciences (IBM-SPSS V 24.0).

Descriptive statistics were calculated to report on the characteristics of the participants, including mothers and children, as well as the main variables of the study, using frequencies, means, and standard deviations. The percentile thresholds was also used to describe the mothers’ level of difficulty in emotional dysregulation. After examining the assumptions (normality, linearity, multicollinearity, and homoscedasticity) of multiple linear regression, which indicated that there were no violations, the regression was conducted. A *P*-value of less than 0.05 was considered statistically significant. To examine the moderating effect of emotional dysregulation on the relationship between anxiety and depression and high caregiving intensity, controlling for socio-demographic factors (mother’s age, working status, educational level, marital status), 2 models of 3-step multiple hierarchical regression analysis were performed. In step 1, socio-demographic characteristics had been entered; in step 2, high caregiving intensity and centered difficulty in emotional regulation (C-DERS) had been entered; and in step 3, the interaction between high caregiving intensity and C-DERS was entered. Anxiety and depression were the dependent variables in the models.

## Results

### Demographic characteristics

Mothers’ characteristics: a total of 192 mothers completed the survey. The analysis (see Table 1). The mean age of the mothers was 33.94 years (SD = 6.89), ranging from 20 to 53 years. Most of the mothers were married (98.40%, *n* = 189). The majority (83.90%, *n* = 161) were not employed. Mothers with a secondary level of education represented 47.90% (*n* = 92). In terms of health, 16.70% (*n* = 32) reported having at least one chronic physical illness such as hypertension, diabetes mellitus, hypothyroidism, migraine, lumbar disc herniation, or irritable bowel syndrome. Furthermore, 24.50% (*n* = 47) of mothers had more than one child diagnosed with LLC and 22.9% (*n* = 44) had high caregiving intensity.

**Table 1.** Descriptive characteristics of mothers (*N* = 192)

Variable		*n*	(%)	M	SD	Med	Min	Max	Q_1_	Q_3_
Age				33.94	6.89	33	20	53	29	39.7
Working tatus	Full time	18	9.4							
	Part time	12	6.3							
	Have not job	161	83.9							
	Retied	1	.5							
Educational level	Basic	30	15.6							
	Secondary	92	47.9							
	Diploma	30	15.6							
	BCs	36	18.8							
	Postgraduate	4	2.1							
Physical lllness	Yes	32	16.7							
	No	160	83.3							
Having another child with LLCs	Yes	47	24.5							
	No	145	75.5							
High caregiving intensity	Yes	44	22.9							
	No	148	77.1							

Q1: quartile 1 = percentile 25, Q3: quartile 3 = percentile 75.

Children’s characteristics: the analysis (see Table 2) showed that the mean age of children was 6.94 years (SD = 4.19), ranging from 1 to 17 years. The majority of children were female, 63% (*n* = 121). Regarding the duration of the diagnosis, it ranged from 1 month to 17 years, with a mean of 3.8 years (SD = 3.54). The cases include different disease categories: neurology at 35.40% (*n* = 68), nephrology at 9.4% (*n* = 18), hematology at 18.20% (*n* = 35), cardiac at 2.10% (*n* = 4), Metabolic at 25.50% (*n* = 49), and other diseases at 9.40% (*n* = 18). Additionally, the cost of treatment ranged from zero to 3.000 JD per year, with a mean of 338.60 JD, and from zero to 30.0 admissions per year, and the mean = 4.00 mean of 4.00 admissions.

**Table 2.** Descriptive characteristics of children (*N* = 192)

Variable		*n*	(%)	M	SD	Med	Min	Max	Q_1_	Q_3_
Age				6.94	4.19	7	1	17	3	11
Duration of illness				3.8	3.5	3	.1	17	1	5
Cost of treatment				338.6	445.3	110	0	3000	100	500
LLC types	Neurology	68	35.40							
	Nephrology	18	9.40							
	Hematology	35	18.20							
	Metabolic	49	25.50							
	Cardiac	4	2.10							
	Others	18	9.40							
Child sex	Male	71	37							
	Female	121	63							
	No	145	25.50							

LLCs: Life-limiting conditions.

## Variables of the study

Difficulty in emotional regulation: The analysis (see Table 3) showed that the total mean score for the whole scale was 41.42 (SD = 16.80), ranging from 16 to 80, with a median of 37.50. Mothers’ scores of DERS were categorized according to percentile thresholds. Scores were divided into 4 levels: the first quartile (<P25), the lowest; the second quartile (between P25 and P50); the third quartile (between P50 and P75); and the fourth quartile (<P75), which is the highest quartile of DERS scores. The analysis showed that 50.00% (*n* = 96) of mothers had a score between 16.0 and 37.0. In addition, 26.6% (*n* = 51) were within the lowest quartile, 23.4% (*n* = 45) were in the second quartile, 26.00% (*n* = 50) were in the third quartile, and 24.00% (*n* = 46) were in the highest quartile of difficulty in emotional regulation scale scores.

**Table 3.** Descriptive statistics of the main variables of the study (*N* = 192)

Variable		*n*	(%)	M	SD	Med	Min	Max	Q_1_	Q_3_
DERS				41.42	16.80	37.5	16	80	27	54
Depressive symptoms				18.29	10.75	18	0	42	10	26
	Normal	31	16.10							
	Mild	14	7.30							
	Moderate	41	21.40							
	Severe	15	7.80							
	Extremely Severe	91	47.40							
Anxiety symptoms				18.63	11.67	17	0	42	10	28
	Normal	37	19.30							
	Mild	5	2.60							
	Moderate	37	19.30							
	Severe	30	15.60							
	Extremely Severe	83	43.20							

DERS: Difficulties in Emotion Regulation Scale.

Anxiety and depression: the analysis of the levels of anxiety and depressive symptoms among the mothers were scored according to the scoring and severity interval guideline of DASS-21 (see Table 3). The mean of anxiety level was 18.63 (SD = 11.67), ranging from zero to 42, with a median = 17. The results showed that 19.30% (*n* = 37) complained of moderate anxiety, 15.60% (*n* = 30) complained of severe, and 43.20% (*n* = 83) complained of extremely severe. In comparison, the mean of depressive symptoms was 18.29 (SD = 10.75), ranging from zero to 42, with a median = 18.0. The results indicated that 21.40% (*n* = 41) had moderate depressive symptoms, 7.80% (*n* = 15) severe, and 47.40% (*n* = 91) extremely severe. The results suggested that the majority of mothers have moderate to extremely severe symptoms of both anxiety and depressive symptoms; 32.20% (*n* = 62) of the mothers experience extremely severe levels of anxiety and depressive symptoms. Symptoms’ severity categorization, including labels such as (extremely severe), was based on validated threshold cutoff points of the DASS-21, which is used as a screening indicator of symptom severity. Therefore, these categorizations reflected the severity of self-reported emotional symptoms. However, it did not reflect the clinical diagnosis of anxiety and depression disorders.

The current study showed good internal consistency for both sub-scales. For the depression sub-scale, Cronbach’s α = 0.87, and the anxiety subscale, Cronbach’s α = 0.89. These findings are consistent with previously published psychometric evidence for DASS-21 (Ali et al. 2017; Alharbi and Osman 2023; Al–Dassean and Murad 2024).

## Testing the moderating effect of emotional dysregulation on the relationship between depression and high caregiving intensity

The results (see Table 4) showed that Model 1. A 3-step hierarchical multiple regression was conducted to examine the emotional dysregulation moderation effect on the relationship between high caregiving intensity and depressive symptoms. In Step 1, sociodemographic (marital status, educational level, work status, mother’s age) variables explained 4.1% of the variance in depressive symptoms. This proportion was not significant (
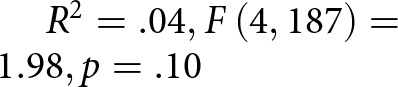
), indicating that these factors do not significantly explain the variance in depressive symptoms. However, the mother’s age had emerged as a significant negative predictor (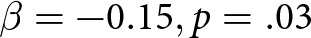) of depressive symptoms. In Step 2, high caregiving intensity and centered emotional dysregulation were added simultaneously; these variables significantly improved the model, Δ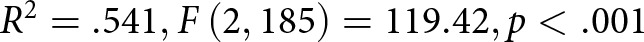, DERS emerging as a strong predictor (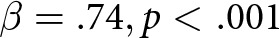), while high caregiving intensity was a positive but non-significant predictor (β = 0.08, *P* = .08) of depressive symptoms. Step 3, the interaction term (DERS × burden) was added; it did not explain additional variance, Δ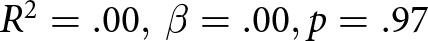, indicating that emotional dysregulation did not moderate the relation between depressive symptoms and caregiving burden. The results of this model suggest that mothers who have higher levels of emotional dysregulation are more likely to have depressive symptoms, irrespective of the presence of high caregiving intensity.

**Table 4.** Three steps – multiple hierarchical regression analysis-testing the moderating effect of emotional dysregulation on the relationship between depressive and anxiety symptoms and caregiving burden, controlling for sociodemographic characteristics among mothers caring for children with life-limiting conditions in Jordan (*N* = 192)

	Model 1		Model 2		Model 3	
Variables	β	*P*- value	β	*P*- value	β	*P*- value
Depression						
Mother’s age	−0.15	0.03	−0.05	0.26	−0.05	0.26
Educational level	0.09	0.24	0.02	0.61	0.02	0.61
Working status	0.00	0.93	0.00	0.95	0.00	0.95
Marital status	0.11	0.12	0.03	0.43	0.03	0.43
Caregiving burden			0.08	0.08	0.08	0.09
Difficulty in emotional regulation			0.73	0.00	0.74	0.00
DERS*Caregiving burden					0.00	0.97
Model	1		2			3
*R^2^*	0.04		0.58			0.58
*Adjusted R^2^*	0.02		0.56			0.56
*R^2^ change*	0.04		0.54			0.00
Anxiety						
Mother’s age	−0.15	0.03	−0.05	0.25	−0.05	0.24
Educational level	0.08	0.29	0.03	0.50	0.03	0.56
Working status	0.04	0.56	0.03	0.29	0.03	0.48
Marital status	0.15	0.03	0.08	0.07	0.08	0.08
Caregiving burden			0.12	0.00	0.13	0.00
Difficulty in emotional regulation			0.73	0.00	0.55	00
DERS*Caregiving Burden					0.18	0.34
Model	1		2		3	
*R*^2^	0.05			0.60	0.60	
Adjusted *R^2^*	0.03			0.58	0.58	
*R^2^* change	0.05			0.54	0.00	

*Denotes multiblue.

## Testing the moderating effect of emotional dysregulation on the relationship between anxiety and high caregiving intensity

A three-step hierarchical multiple regression was conducted to examine the moderating effect of emotional dysregulation on the relationship between anxiety and caregiving burden. Step 1 included sociodemographic variables (marital status, educational level, work status, and mother’s age), which explained a small proportion of the variance in anxiety symptoms (5.3%), (R^2^ = 05, F (4,187) = 2.62, *P* = 0.03). Mother’s age (β = − 0.15, *P* = 0.03) revealed that older mothers report a slightly lower level of anxiety and marital status (β = 0.16, *P* = 0.03) were significant predictor of mothers’ level of anxiety. In Step 2, high caregiving intensity and centered emotional dysregulation were added, and these variables showed a significant increase in explained variance, ΔR^2^ = 54, F (2,185) = 126.71, *P* < 0.001. The emotional dysregulation (β = 0.74, *P* < 0.001) and high caregiving intensity (β = 0.13, *P* = 0.007) were significant predictors of anxiety symptoms. In step 3, the interaction term was added, and there was no significant ΔR^2^ = 0.00, *P* = 0.34. This indicated that emotional dysregulation did not moderate the relationship between anxiety symptoms and caregiving burden. However, high caregiving intensity and emotional dysregulation were 2 strong independent predictors of anxiety symptoms among mothers caring for children with life-limiting conditions.

## Discussion

Mental well-being of mothers caring for children with life-limiting conditions is an essential component of their willingness to care for their children. Their psychological resources are assumed to influence their ability to manage the burden due to the sickness of their children. Consequently, they will be able to provide continuous, effective, and supportive care for their children. However, the dynamic process and the complexity of care may contribute to an increased caregiving burden, which in turn can exacerbate psychological distress. The current study examined the moderating effect of emotional dysregulation on the relationship between high caregiving intensity and anxiety and depressive symptoms among mothers caring for children with life-limiting conditions. The results indicated that emotional dysregulation did not significantly moderate the relationship between high caregiving intensity and anxiety or depressive symptoms. This indicates that the strength of the relationship between high caregiving intensity, anxiety, and depressive symptoms did not differ across the levels of emotional dysregulation among mothers caring for children with life-limiting conditions. The findings sustain the notion that while emotional dysregulation does not moderate the relationship between high caregiving intensity and severity of depressive and anxiety symptoms, it is proposed that emotional dysregulation is associated directly with these factors rather than serving as a modifier or moderator. This adds to the body of knowledge that the effect of emotional dysregulation depends on several issues, including the cultural context, the severity of symptoms, and the perception of mothers regarding components of emotional regulation itself.

One possible explanation for the non-significant moderating effect of emotional dysregulation is that burden, anxiety, and depression are influenced primarily by cognitive components, rather than by emotional processes alone. This interpretation is supported by the significant direct associations observed between emotional dysregulation and these outcomes. In other words, while components of emotional dysregulation were significantly associated with the severity of burden, anxiety, and depression, emotional dysregulation lacks sufficient cognitive elements to alter the strength of these relationships. Consequently, its effect was not statistically sufficient to produce a significant moderating role. Furthermore, the higher high caregiving intensity may gradually destabilize mothers’ capacity to normalize emotional responses, as assessed by validated difficulties on the DERS, which, in sequence, heightens vulnerability to anxiety and depressive symptoms (Gratz and Roemer 2004; Hallion et al. 2018; Colonnello et al. 2024). In such a model, emotional dysregulation had not altered the strength of the burden–psychological distress relationship; rather, it serves as a proximal pathway linking the caregiving burden to its psychological distress (Gratz and Roemer 2004). This interpretation is consistent with the transdiagnostic model that position emotion–dysregulation as a main mechanism connecting chronic stress experience to internalizing symptoms (Hallion et al. 2018; Zaid et al. 2025).

DBT views emotion dysregulation as an outcome of the interaction between biological vulnerability and unsupportive environments, which is especially relevant for mothers of children with life-limiting conditions. This leads to pervasive emotional reactivity (Linehan et al. 2015). Mothers who struggle with emotional regulation often have difficulty identifying their feelings or applying regulation strategies, resulting in intense emotional responses to caregiving stressors (Linehan, Bohus, & Lynch 2007; Linehan et al. 2015). The dialectical approach of balancing acceptance and change promotes an adaptive mindset: mothers with emotional regulation skills learn to accept their emotional burdens while actively developing skills to reduce distress and modify their emotional responses (Linehan 2015). Depression is usually connected with persistent rumination and difficulties in initiating cognitive reappraisal, while anxiety relates to worries and avoidance (Blanke et al. 2021). Therefore, even with the absence of a moderation effect, emotional dysregulation remains clinically relevant. The results of this study indicate that emotional dysregulation is associated with higher levels of anxiety and depressive symptoms, highlighting its role as a potential intervention target. Interventions aimed to improve emotional regulation skills may help reduce psychological distress among mothers caring for children with LLCs, regardless of the level of caregiving burden.

In addition, a non-significant moderation effect may be related to conceptual and methodological considerations. Dichotomization of the high caregiving intensity reduced the score variability and statistical power. In addition, emotional dysregulation may be more related to subjective high caregiving intensity than the objective score that was used in this study.

The findings of this study also showed that mothers experienced extremely severe levels of anxiety and depressive symptoms. This suggests that they are facing difficulties in starting activities, dealing with persistent negative emotions, engaging in psychological rumination, and feeling sad. While such levels are expected based on existing research on mothers of children with chronic and debilitating health conditions who do not receive treatment, being at a severe level is an alarming sign of their psychological well-being. The high level of anxiety indicates that mothers are suffering from physical and psychological symptoms such as a dry throat, trembling hands, palpitations, crying, insomnia, unexplained fears, overthinking, and worries. These symptoms may be linked to their inability to control and manage their child’s disease symptoms, fears of imminent death, concerns about having another child with the same illness, and other family responsibilities. Although increased anxiety and depression are common consequences of high caregiving intensity s, developing emotional regulation skills could help mitigate these negative effects, highlighting the potential of targeting DER to reduce the severity of these symptoms. The importance of focusing on ER stems from the fact that many mothers in this study struggle with the regulatory skills needed to manage ongoing emotional challenges, as they navigate accepting their child’s condition while holding onto unrealistic hopes for a cure. These findings align with previous research showing significantly higher rates of anxiety and depression among mothers of children with LLCs compared to those with healthy children or children with other chronic conditions (Fraser et al. 2021; Lewandowska et al. 2024). However, mothers in this study exhibited even more severe symptoms, exceeding those reported in earlier research. This may be due to factors such as the lack of comprehensive palliative care services in Jordan for these children and their families, leading to limited psychological support (Hamdan et al. 2023), and the use of different assessment tools with varying cutoff points in previous studies. Additionally, most mothers in this study were unemployed, spending much of their time at home caring for their children, which reduced their social engagement and financial independence, both well-known risk factors for increased anxiety and depression (Liang et al. 2023). Furthermore, in Arab culture, mothers, who are the primary caregivers, are presumed to bear the biopsychosocial and emotional responsibilities associated with their roles, as social expectations and cultural traditions dictate (Ghannam et al. 2017), which exacerbates the feeling of inadequacy and worsens the psychological distress. Sickness among most of the Arab Muslim community is partially assumed to be a test from God, which requires them to be patient and tolerate the extreme burden of the disease process and the caring aspect of the sick child (Rossiter et al., 2019) This implies that for those with children with chronic illness and LLCs, caring is a family crisis leading to extreme levels of bio-psychosocial burden on the primary caregiver, which is primarily the mothers when the sick person in the family is the child. This could explain the very high levels of anxiety and depression observed.

The high levels of anxiety and depression among mothers due to the constant burden of caregiving and high levels of emotional dysregulation should alert the health care professionals to the affective role of caregiving of children with LLCs, and the need to have both mothers and their children included in psychological counselling programs that enhance their emotional and psychological resources. Incorporating DBT-based emotional regulation interventions provide structured pathway to enhanced psychological resources of the mothers.

However, a cross-sectional design offers only descriptive correlation at a single point in time; causality relationships cannot be inferred in this study. Therefore, future longitudinal studies are needed to examine the mediating effect of emotional dysregulation and the ongoing dynamics of the study variables among mothers of children with LLCs. In addition, while this study controlled for socioeconomic variables, other factors such as family structure, social support, and financial status could also be explored. Finally, although high caregiving intensity is operationalized as practical indicators, this may limit the study’s comparability with other studies and limit the exploration of mothers’ self-perception of burden.

## Conclusion

The current study emphasizes that emotional dysregulation did not function as a moderator, but rather predicted and associated with high levels of anxiety and depression. In practice, this suggests that psychosocial care could target emotion regulation capacities, building skills in emotional awareness, acceptance, cognitive reappraisal, and adaptive coping, and integrating these modules into routine caregiver services to alleviate psychological distress. To extend this study, future longitudinal conditional process research designs recommend conducting to establish causal pathways (burden → dysregulation → symptoms), conducting intervention trials that examine emotion regulation–intervention within pediatric palliative contexts. The intervention should be culturally tailored and incorporate additional cognitive components that are more directly relevant to depression and anxiety in the target populations, taking into account their specific medical and psychological conditions.
